# Modeling of a Novel T-Core Sensor with an Air Gap for Applications in Eddy Current Nondestructive Evaluation

**DOI:** 10.3390/s24247931

**Published:** 2024-12-11

**Authors:** Siquan Zhang

**Affiliations:** Department of Electrical and Automation, Shanghai Maritime University, Shanghai 201306, China; sqzhang@shmtu.edu.cn

**Keywords:** eddy current testing, ferrite-core coil, T-core coil sensor, analytical model, truncated region eigenfunction expansion method

## Abstract

Multi-layer conductive structures, especially those with features like bolt holes, are vulnerable to hidden corrosion and cracking, posing a serious threat to equipment integrity. Early defect detection is vital for implementing effective maintenance strategies. However, the subtle signals produced by these defects necessitate highly sensitive non-destructive testing (NDT) techniques. Analytical modeling plays a critical role in both enhancing defect-detection capabilities and guiding the design of highly sensitive sensors for these complex structures. Compared to the finite element method (FEM), analytical approaches offer advantages, such as faster computation and high accuracy, enabling a comprehensive analysis of how sensor and material parameters influence defect detection outcomes. This paper introduces a novel T-core eddy current sensor featuring a central air gap. Utilizing the vector magnetic potential method and a truncated region eigenfunction expansion (TREE) method, an analytical model was developed to investigate the sensor’s interaction with multi-layer conductive materials containing a hidden hole. The model yielded closed-form expressions for the induced eddy current density and coil impedance. A comparative study, implemented in Matlab, analyzed the eddy current distribution generated by T-core, E-core, I-core, and air core sensors under identical conditions. Furthermore, the study examined how the impedance of the T-core sensor changed at different excitation frequencies between 100 Hz and 10 kHz when positioned over a multi-layer conductor with a hidden air hole. These findings were then compared to those obtained from E-core, I-core, and air-core sensors. The analytical results were validated through finite element simulations and experimental measurements, exhibiting excellent agreement. The study further explored the influence of T-core design parameters, including the air gap radius, dome radius, core column height, and relative permeability of the T-core material, on the inspection sensitivity. Finally, the proposed T-core sensor was used to evaluate crack and hole defects in conductors, demonstrating its superior sensitivity compared to I-core and air core sensors. Although slightly less sensitive than the E-core sensor, the T-core sensor offers advantages, including a more compact design and reduced material requirements, making it well-suited for inspecting intricate and confined surfaces of the target object. This analytical model provides a valuable tool for designing advanced eddy current sensors, particularly for applications like detecting bolt hole defects or measuring the thickness of non-conductive coatings in multi-layer conductor structures.

## 1. Introduction

Multi-layer metal structures, crucial for equipment like aircraft skins, oil pipelines, and nuclear power plant heat exchangers, are susceptible to safety hazards posed by cracks, corrosion, and other hidden defects [1]. Over time, these structures inevitably develop corrosion and fatigue cracks, leading to thinning and compromised structural integrity [2,3]. These defects can negatively impact equipment performance and safety, highlighting the need for early detection to prevent catastrophic consequences.

To ensure safety, crucial equipment components are regularly inspected using NDT methods. Common NDT methods include liquid penetrant testing, magnetic particle testing, ultrasonic inspection, eddy current testing (ECT), radiography, and infrared thermography [4,5]. While liquid penetrant testing is fast and easy, it only detects surface defects and requires extensive surface cleaning. Magnetic particle testing is limited to ferromagnetic materials and necessitates demagnetization. Ultrasonic flaw detection is effective for identifying deep defects in thick conductors and can also reveal internal flaws in multi-layer conductor configurations [6,7]. However, it typically requires a uniform and well-filled adhesive or sealant layer between these multi-layer conductors. Traditional ultrasonic testing methods struggle when it comes to detecting multi-layer structures that have air gaps between the conductor layers [8,9,10]. Infrared thermography, while useful, relies on complex equipment and trained operators. ECT, a widely used and effective technique, is particularly well-suited for detecting defects in multi-layer conductive structures, especially surface and subsurface micro-cracks and corrosion [11]. This non-contact method offers high sensitivity due to its ability to detect changes in eddy currents induced within the conductor by an alternating current excitation. These eddy currents generate a secondary magnetic field, which is influenced by factors like the sensor–conductor distance, material properties, and the presence of defects. Any change in the magnetic field, resulting from defect-induced variations in the eddy currents, alters the impedance of the excitation coil or the induced voltage in a receiving coil. This sensitivity makes ECT applicable to a wide range of applications, including distance measurements, material property assessments, and coating thickness determinations.

While effective, traditional ECT suffers from limitations, such as the shallow penetration depth of high-frequency eddy currents. While lower frequencies improve penetration, they compromise sensitivity. To address this, emerging magnetic sensing technologies, including Hall sensors, Giant Magnetoresistive (GMR) sensors, and Tunnel Magnetoresistive (TMR) sensors [12,13,14], are increasingly integrated with traditional ECT excitation coils. These advanced sensors greatly enhance ECT’s sensitivity and accuracy. GMR sensors leverage the giant magnetoresistance effect to detect minute variations in magnetic fields [15,16], providing high sensitivity and resolution. For example, Joseph utilized a high-current, low-frequency excitation coil and a GMR sensor array to quantify corrosion defects in a pipe without removing the insulation [17]. TMR sensors, employing quantum tunneling phenomena, demonstrate even greater sensitivity than GMR, making them ideal for high-performance applications. Betta developed a novel ECT probe with a double coil excitation and a triaxial TMR sensor array, achieving high signal-to-noise ratios for thin defect detection [18]. Fei Yang et al. designed a flexible eddy current TMR (FEC-TMR) sensor and successfully detected internal cracks in metal joints [19].

Dodd and Deeds pioneered the use of Bessel functions to model electromagnetic fields generated by coils interacting with conductive materials [20]. This approach, employing Bessel function series, enabled the analysis of various coil geometries and material configurations, such as coil–plane, coil–tube, and coil–rod systems. It allowed for the accurate prediction of ECT signals by expressing electromagnetic fields and impedance changes as Bessel function series, ultimately resulting in an integral solution. Subsequently, Theodoulidis developed the TREE method [21]. This technique employs domain truncation, eigenfunction expansion, and the matching of boundary and surface conditions, providing a flexible and efficient solution for problems involving intricate geometries and boundary conditions [22]. In contrast to conventional ECT methods, the TREE method yields a series solution, resulting in faster computation and the ability to control accuracy by adjusting the number of series terms included.

Improving defect detection sensitivity in multi-layer conductors involves directing the excitation sensor’s magnetic flux along low-resistance paths [5,23,24]. This increases magnetic flux penetration into the conductor, enabling deeper eddy current penetration and the enhanced detection of deeper defects. Ferrite cores, composed of iron oxide and metallic additives, exhibit tunable magnetic properties [25,26]. Their high permeability and low loss at high frequencies make them ideal for coil sensors that operate through electromagnetic induction. The TREE method [27,28], initially applied to model simple sensor–conductor interactions in ferrite core ECT sensors, has evolved to address more complex scenarios. This includes analyzing eddy current problems involving intricate conductor defect geometries and various core configurations, such as the E-core, C-core, I-core, and T-core [22,29]. Extensive research has focused on E-core and I-core sensors with air gaps [30,31], as well as T-core sensors without air gaps [32]. Ferrite cores, owing to their flux concentration and shielding properties, enhance the flux density and sensor sensitivity compared to the air-core. The sensitivity of an ECT sensor varies depending on the specific ferrite core configuration and size, highlighting the importance of considering both sensitivity and core characteristics during sensor selection. Despite these advancements, accurately locating hidden defects within multilayer structures using ECT sensors remains a significant challenge.

This paper presents the first application of the TREE method to analyze a novel T-core ECT sensor. The sensor features a circular air gap positioned above a conductive layer containing a hidden hole. A homogeneous Dirichlet boundary condition is applied to the truncated surface, with the azimuthal component of the magnetic vector potential (A_φ_) expressed as a series of orthogonal eigenfunctions. By carefully selecting eigenfunctions and applying field continuity conditions at boundaries and interfaces, the series coefficients and eigenvalues are determined. Truncating the infinite solution domain to a finite space allows for a series solution instead of an integral, resulting in a faster numerical calculation and easier error control. The analysis begins with a filamentary coil encircling the T-core column, deriving expressions for the magnetic vector potential. This analysis is then extended to coils with rectangular cross-sections using the superposition method to obtain the magnetic vector potential in each region. Finally, closed-form expressions for the induced eddy current density and coil impedance of the T-core ECT sensor are derived. The analytical model provides a valuable tool for evaluating the ECT sensor, allowing for the analysis of individual parameters. The T-core sensor’s coil impedance variation is calculated for frequencies ranging from 100 Hz to 10 kHz. These results are compared with those from E-core, I-core, and air-core sensors. Further validation is achieved through a comparison with FEM simulations and experimental results, showing good agreement. The results highlight the significantly higher sensitivity of the T-core ECT sensor compared to I-core and air-core sensors when detecting hidden defects in a multi-layer conductor. Analytical and simulation methods are used to analyze the eddy current distribution produced by different core sensors (E, T, I, and air) under identical excitation conditions, providing insights into the T-core’s superior sensitivity. The influence of key T-core parameters on sensor sensitivity is also assessed, leading to the determination of optimal sensor dimensions.

## 2. Analysis

**Figure 1:**
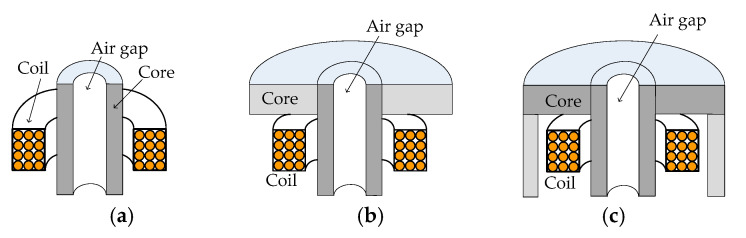
Cross-sectional illustrations of (**a**) an I-core, (**b**) a T-core, and (**c**) an E-core ECT sensors, each featuring an air gap within the ferrite core.

Figure 1 illustrates three ferrite core ECT sensor types: I-core, T-core, and E-core. These sensors share a common structural feature, a circular air gap within the core column, and display structural similarities. Notably, the E-core can be derived from the T-core by adding a cylindrical shield, while the I-core results from removing the T-core’s upper ring plane [33].

However, the T-core sensor with its air gap (Figure 1b) has not yet been subjected to theoretical or experimental analysis. Consequently, its sensitivity and other performance characteristics require investigation and a comparison with the E-core and I-core ECT sensors [34].

**Figure 2:**
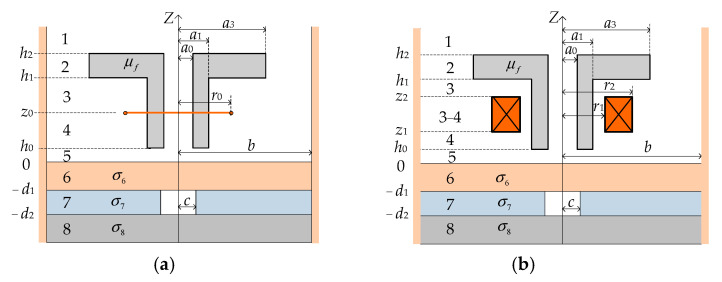
T-core ECT sensors consisting of (**a**) a filamentary coil and (**b**) a multi-turn coil positioned above a layered conductor with a hole in its second layer. Numbers 1–8 represent different regions.

Figure 2a illustrates the initial configuration for analysis, featuring a filamentary coil driven by a harmonic current, *Ie^jωt^*. This configuration involves a sensor positioned above a non-magnetic, conducting half-space comprised of three layers with conductivities σ_6_, σ_7_, and σ_8_. The second layer contains an air hole. The *z* = 0 plane corresponds to the upper surface of this multi-layered conductor, resulting in a problem domain with eight distinct regions. The method of variable separation is employed, and the resulting expressions for each region depicted in Figure 2a are presented in matrix form below.

(1) $$A_{1}(r,z)=J_{1}(q^{T}r)q^{-1}e^{-qz}C_{1}$$

(2) $$A_{2}(r,z)=\left\{\begin{array}{c} J_{1}(m^{T}r)\\ R_{1}(m^{T}r)\\ R_{1}^{\prime }(m^{T}r) \end{array}m^{-1}(e^{-mz}C_{2}-e^{mz}B_{2})\begin{array}{ccc} & 0\leq r\leq a_{0} & \\ & a_{0}\leq r\leq a_{3} & \\ & a_{3}\leq r\leq b & \end{array}\right.$$

(3) $$A_{3}(r,z)=\left\{\begin{array}{c} J_{1}(p^{T}r)\\ L_{1}(p^{T}r)\\ L_{1}^{\prime }(p^{T}r) \end{array}p^{-1}(e^{-pz}C_{3}-e^{pz}B_{3})\begin{array}{ccc} & 0\leq r\leq a_{0} & \\ & a_{0}\leq r\leq a_{1} & \\ & a_{1}\leq r\leq b & \end{array}\right.$$

(4) $$A_{4}(r,z)=\left\{\begin{array}{c} J_{1}(p^{T}r)\\ L_{1}(p^{T}r)\\ L_{1}^{\prime }(p^{T}r) \end{array}p^{-1}(e^{-pz}C_{4}-e^{pz}B_{4})\begin{array}{ccc} & 0\leq r\leq a_{0} & \\ & a_{0}\leq r\leq a_{1} & \\ & a_{1}\leq r\leq b & \end{array}\right.$$

(5) $$A_{5}(r,z)=J_{1}(q^{T}r)q^{-1}(e^{-qz}C_{5}-e^{qz}B_{5})$$

(6) $$A_{6}(r,z)=J_{1}(q^{T}r)s_{6}^{-1}(e^{-s_{6}z}C_{6}-e^{s_{6}z}B_{6})$$

(7) $$A_{7}=\begin{array}{c} F_{1}(vc)J_{1}(u^{T}r)\\ F_{1}(v^{T}r)J_{1}(uc) \end{array}u^{-1}(e^{-uz}C_{7}-e^{uz}B_{7})\begin{array}{ccc} & \begin{array}{c} 0\leq r\leq c\\ c\leq r\leq b \end{array} & \end{array}$$

(8) $$A_{8}(r,z)=-J_{1}(q^{T}r)s_{8}^{-1}e^{s_{8}z}B_{8}$$

where $s_{6}=\sqrt{q^{2}+j\omega \mu_{0}\mu_{r6}\sigma_{6}}$, $s_{8}=\sqrt{q^{2}+j\omega \mu_{0}\mu_{r8}\sigma_{8}}$.

The analytical solution is derived using Bessel functions of the first and second kind, denoted by *J_n_* and *Y_n_*, respectively, where *n* represents the order of the function. The parameters $J_{1}(q^{T}r)$, $J_{1}(m^{T}r)$, $R_{1}(m^{T}r)$, $R_{1}^{\prime }(m^{T}r)$, $J_{1}(p^{T}r)$, $L_{1}(p^{T}r)$, $L_{1}^{\prime }(p^{T}r)$, $J_{1}(u^{T}r)$, and $F_{1}(v^{T}r)$ are represented by row vectors. Additionally, $q^{-1}$, $m^{-1}$, $p^{-1}$, $u^{-1}$, $v^{-1}$, $s_{6}^{-1}$, $s_{8}^{-1}$, and the exponential functions are expressed as diagonal matrices. Finally, $C_{k}$ and $B_{k}$ (where *k* ranges from 1 to 8, representing different regions) are column vectors of unknown coefficients.

Figure 2’s regions 1, 5, 6, and 8 have eigenvalues (*q_i_*) that are the positive real roots of Equation (9), where *i* represents the root number.

(9) $$J_{1}(q_{i}b)=0$$

Region 2 is further divided into three subregions. Subregions I (0 ≤ *r* ≤ *a_0_*) and III (*a_3_* ≤ *r* ≤ b) are filled with air, while subregion II (*a_0_* ≤ *r* ≤ *a_3_*) contains a ferrite core. The positive real roots of Equation (10) yield the discrete eigenvalues (*m_i_*).

(10) $$R_{1}^{\prime }(m_{i}b)=0$$

where

(11) $$R_{n}^{\prime }(m_{i}r)=B_{3F}J_{n}(m_{i}r)+C_{3F}Y_{n}(m_{i}r)$$

(12) $$C_{3F}=\frac{\pi m_{i}a_{3}}{2}[\frac{J_{1}(m_{i}a_{3})R_{0}(m_{i}a_{3})}{\mu_{f}}-J_{0}(m_{i}a_{3})R_{1}(m_{i}a_{3})]$$

(13) $$B_{3F}=\frac{\pi m_{i}a_{3}}{2}[Y_{0}(m_{i}a_{3})R_{1}(m_{i}a_{3})-\frac{Y_{1}(m_{i}a_{3})R_{0}(m_{i}a_{3})}{\mu_{f}}]$$

(14) $$R_{n}(m_{i}r)=B_{2F}J_{n}(m_{i}r)+C_{2F}Y_{n}(m_{i}r)$$

(15) $$C_{2F}=\frac{\pi m_{i}a_{0}}{2}J_{0}(m_{i}a_{0})J_{1}(m_{i}a_{0})(\mu_{f}-1)$$

(16) $$B_{2F}=\frac{\pi m_{i}a_{0}}{2}[J_{1}(m_{i}a_{0})Y_{0}(m_{i}a_{0})-J_{0}(m_{i}a_{0})Y_{1}(m_{i}a_{0})\mu_{f}]$$

Regions 3 and 4 are subdivided into three subregions: Subregion I (0 ≤ *r* ≤ *a_0_*), Subregion II (*a_0_* ≤ *r* ≤ *a_1_*), and Subregion III (*a_1_* ≤ *r* ≤ *b*). Subregions I and III represent air, while Subregion II encompasses the ferrite core. The discrete eigenvalues, denoted by *p*_i_, are positive real roots of Equation (17):

(17) $$L_{1}^{\prime }(p_{i}b)=0$$

where

(18) $$L_{n}^{\prime }(p_{i}r)=B_{3F}^{\prime }J_{n}(p_{i}r)+C_{3F}^{\prime }Y_{n}(p_{i}r)$$

(19) $$C_{3F}^{\prime }=\frac{\pi p_{i}a_{1}}{2}[\frac{J_{1}(p_{i}a_{1})L_{0}(p_{i}a_{1})}{\mu_{f}}-J_{0}(p_{i}a_{1})L_{1}(p_{i}a_{1})]$$

(20) $$B_{3F}^{\prime }=\frac{\pi p_{i}a_{1}}{2}[Y_{0}(p_{i}a_{1})L_{1}(p_{i}a_{1})-\frac{Y_{1}(p_{i}a_{1})L_{0}(p_{i}a_{1})}{\mu_{f}}]$$

(21) $$L_{n}(p_{i}r)=B_{2F}^{\prime }J_{n}(p_{i}r)+C_{2F}^{\prime }Y_{n}(p_{i}r)$$

(22) $$C_{2F}^{\prime }=\frac{\pi p_{i}a_{0}}{2}J_{0}(p_{i}a_{0})J_{1}(p_{i}a_{0})(\mu_{f}-1)$$

(23) $$B_{2F}^{\prime }=\frac{\pi p_{i}a_{0}}{2}[J_{1}(p_{i}a_{0})Y_{0}(p_{i}a_{0})-J_{0}(p_{i}a_{0})Y_{1}(p_{i}a_{0})\mu_{f}]$$

Region 7 comprises two subregions: an air space (0≤r≤c) and a conductive material (c≤r≤b). Applying the interface condition in the radial direction at *r* = *c* yields the following equation. The eigenvalues, *u_i_*, are then determined as the positive real roots of Equation (24):

(24) $$u_{i}F_{1}(v_{i}c)J_{0}(u_{i}c)=\mu_{r7}^{-1}v_{i}F_{0}(v_{i}c)J_{1}(u_{i}c)$$

where

(25) $$F_{n}(v_{i}r)=J_{n}(v_{i}r)Y_{1}(v_{i}b)-J_{1}(v_{i}b)Y_{n}(v_{i}r)$$

The relationship between eigenvalues (*u_i_*) and their corresponding eigenvectors (*v_i_*) is as follows:

(26) $$u_{i}=\sqrt{v_{i}^{2}+j\omega \sigma_{7}\mu_{0}\mu_{r7}}$$

The eigenvalues (*u_i_*), which are the complex roots of Equation (24), can be determined using the Newton–Raphson method [35,36,37]. While this method is effective, more efficient algorithms have been developed in recent years to ensure the identification of all roots [38,39].

To solve the problem, the interface conditions between the eight regions must be satisfied, specifically the continuity of *B_z_* and *H_r_*. Determining the magnetic vector potential $A_{(k)filamentary}(r,z)$ in each region, excited by the T-core filamentary coil (Figure 2a), requires solving for the discrete eigenvalues and unknown coefficients through these continuity conditions at various boundaries and interfaces. Subsequently, the magnetic vector potential of each region excited by a coil with a rectangular cross-section can be derived using the superposition method.

(27) $$A_{k}^{coil}(r,z)=\int_{r_{1}}^{r_{2}}\int_{z1}^{z_{2}}A_{(k)filamentary}(r,z,r_{0},z_{0})dr_{0}dz_{0},k=1,2,\ldots ,8$$

The eddy current density within the layered conductive material can be determined using the method outlined in [21].

(28) $$J_{L}^{eddy}(r,z)=-j\omega \sigma_{L}A_{L}^{coil}(r,z);\begin{array}{cc} & L=6,7,8 \end{array}$$

The final expression for the eddy current density within the first-layer conductor (region 6) can be derived as follows.

(29) $$\begin{array}{l} J_{6}^{eddy}(r,z)=-j\omega \sigma_{6}A_{6}^{coil}(r,z)\\ =\frac{-j\omega \sigma_{6}\mu NI}{2(r_{2}-r_{1})(z_{2}-z_{1})}J_{1}(qr)s_{6}^{-1}W_{4}W_{2}^{-1}W_{3}p^{-3}D^{-1}\chi (pr_{1},pr_{2}) \end{array}$$

Following the derivation of magnetic vector potentials $A_{3}^{coil}$ and $A_{4}^{coil}$ for regions 3 and 4 in Figure 2b, the potential $A_{3-4}^{coil}$ within the region between these two regions can be determined by substituting *z* for *z_2_* in $A_{3}^{coil}$ and *z* for *z*_1_ in $A_{4}^{coil}$ and summing the results. The final impedance expressions for the T-core coil sensor are then derived as follows:

(30) $$\begin{array}{l} Z=\frac{j\omega 2\pi N}{I(z_{2}-z_{1})(r_{2}-r_{1})}\int_{r_{1}}^{r_{2}}\int_{z_{1}}^{z_{2}}rA_{3-4}^{coil}(r,z)drdz\\ =\frac{j\omega \mu \pi N^{2}}{{\left(r_{2}-r_{1}\right)}^{2}{\left(z_{2}-z_{1}\right)}^{2}}\chi (pr_{1},pr_{2})p^{-4}\\ \cdot [2(z_{2}-z_{1})p+e^{p(z_{1}-z_{2})}-e^{p(z_{2}-z_{1})}+W_{1}W_{2}^{-1}W_{3}]p^{-3}D^{-1}\chi (pr_{1},pr_{2}) \end{array}$$

where

(31) $$\chi (pr_{1},pr_{2})=\int_{pr_{1}}^{pr_{2}}(pr)L_{1}^{\prime }(pr)d(pr)]$$

(32) $$W_{1}=(e^{-pz_{1}}-e^{-pz_{2}})C_{48}-(e^{pz_{2}}-e^{pz_{1}})B_{48}$$

(33) $$W_{2}=(\lambda_{1}F^{-1}G+\lambda_{2}F^{-1}H)e^{-ph_{1}}C_{48}-(\lambda_{1}F^{-1}G-\lambda_{2}F^{-1}H)e^{ph_{1}}B_{48}$$

(34) $$W_{3}=(\lambda_{1}F^{-1}G-\lambda_{2}F^{-1}H)(e^{p(h_{1}-z_{1})}-e^{p(h_{1}-z_{2})})-(\lambda_{1}F^{-1}G+\lambda_{2}F^{-1}H)(e^{p(z_{2}-h_{1})}-e^{p(z_{1}-h_{1})})$$

(35) $$W_{4}=e^{-s_{6}z}C_{68}-e^{s_{6}z}B_{68}$$

(36) $$\lambda_{1}=(T-U)e^{m(h_{1}-h_{2})}+(T+U)e^{m(h_{2}-h_{1})}$$

(37) $$\lambda_{2}=(T-U)e^{m(h_{1}-h_{2})}-(T+U)e^{m(h_{2}-h_{1})}$$

(38) $$C_{48}=\frac{1}{2}e^{ph_{0}}D^{-1}[(H^{*}+G*)e^{-qh_{0}}C_{58}+(H*-G*)e^{qh_{0}}B_{58}]$$

(39) $$B_{48}=\frac{1}{2}e^{-ph_{0}}D^{-1}[(H*-G*)e^{-qh_{0}}C_{58}+(H*+G*)e^{qh_{0}}B_{58}]$$

(40) $$C_{58}=\frac{1}{2}[(\mu_{6}^{-1}+qs_{6}^{-1})C_{68}+(\mu_{6}^{-1}-qs_{6}^{-1})B_{68}]$$

(41) $$B_{58}=\frac{1}{2}[(\mu_{6}^{-1}-qs_{6}^{-1})C_{68}+(\mu_{6}^{-1}+qs_{6}^{-1})B_{68}]$$

(42) $$C_{68}=\frac{1}{2}e^{-s_{6}d_{1}}[(E^{-1}N+s_{6}q^{-1}E^{-1}V)e^{ud_{1}}C_{78}+(E^{-1}N-s_{6}q^{-1}E^{-1}V)e^{-ud_{1}}B_{78}]$$

(43) $$B_{68}=\frac{1}{2}e^{s_{6}d_{1}}[(E^{-1}N-s_{6}q^{-1}E^{-1}V)e^{ud_{1}}C_{78}+(E^{-1}N+s_{6}q^{-1}E^{-1}V)e^{-ud_{1}}B_{78}]$$

(44) $$C_{78}=\frac{1}{2}e^{-ud_{2}}(N^{-1}E-V^{-1}Eqs_{8}^{-1})e^{-s_{8}d_{2}}$$

(45) $$B_{78}=\frac{1}{2}e^{ud_{2}}(N^{-1}E+V^{-1}Eqs_{8}^{-1})e^{-s_{8}d_{2}}$$

where

(46) $$C_{n7}=\frac{C_{n}}{B_{7}}$$

(47) $$B_{n7}=\frac{B_{n}}{B_{7}}$$

The matrices **T**, **U**, **F**, **G**, G*, **H**, H*, **N**, **V**, **D**, and **E** are detailed in Appendix A.

## 3. Numerical Implementation

**Figure 3:**
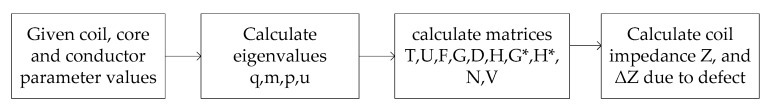
Numerical calculation scheme of the analytical model.

**Figure 4:**
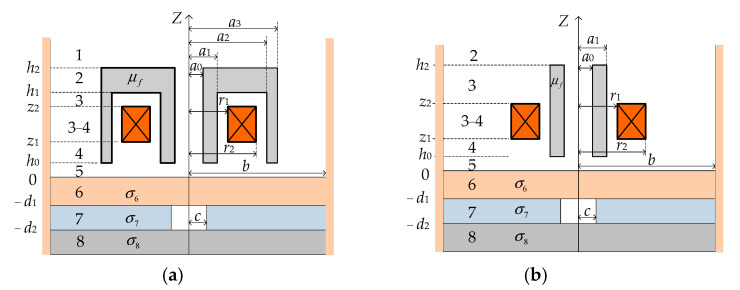
Axially symmetric (**a**) E-core and (**b**) I-core ECT sensors positioned above a layered conductor with a hidden hole in the second layer. Numbers 1–8 represent different regions.

The eddy current density in the first layer conductor and the impedance of the T-core coil were calculated using the Matlab software package version 7.0, based on Equations (29) and (30). The main steps of the numerical calculation procedure are shown in Figure 3. Employing the same derivation method used for the T-core sensor, expressions for the coil impedance of E-core and I-core sensors, along with the eddy current density in the conductor (as depicted in Figure 4), were derived. Subsequently, the coil impedances of the E-core, I-core (with relative permeability *μ_r_* = 2500), and air-core (*μ_r_* = 1) sensors and the corresponding eddy current densities at the same conductor depth were determined using analytical methods.

The analytical calculations utilize parameters derived from measurements of the cored sensors and conductors employed in the experiments, as detailed in Table 1. For all analytical calculations, the solution domain was truncated at *b* = 90 mm (nine times the coil’s outer radius, *r*_2_), and the number of summation terms was set to *N_s_* = 60. A comparative analysis was then conducted, comparing the analytical results with those obtained from finite element analysis and experimental measurements.

## 4. Experimental Confirmation

**Figure 5:**
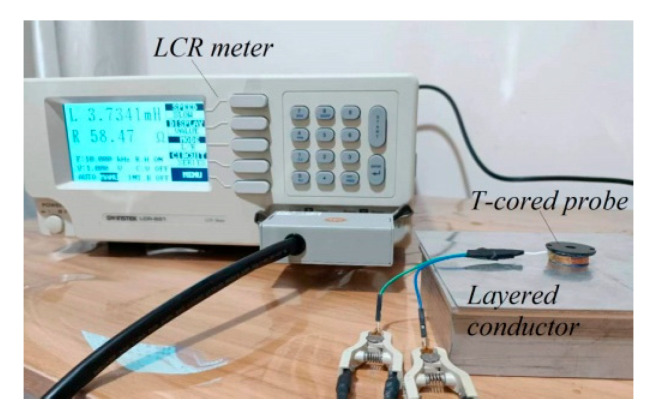
Configuration for the experiment.

**Figure 6:**
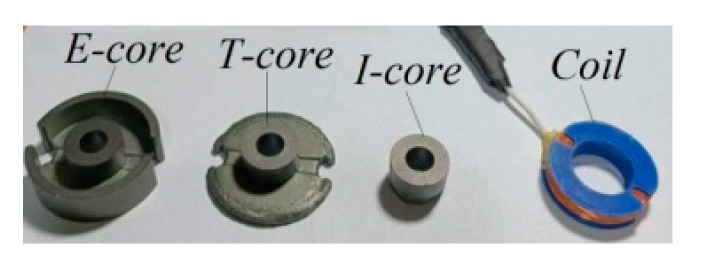
E-core, T-core, I-core, and coil used in the experiments.

The proposed analytical model was validated through experimental verification. The experimental setup, shown in Figure 5, comprised a T-core sensor, a layered conductor, and a Gwinstek LCR meter. Figure 6 displays the E-core, T-core, I-core, and air coil used in the experiments. The parameters detailed in Table 1 also served as inputs for these experimental investigations, which involved measuring impedance changes across a frequency range of 100 Hz to 10 kHz. These impedance changes were observed in each core sensor due to the presence of a layered conductor containing a hidden hole.

## 5. Comparison with FEM

To validate the proposed analytical model, Ansoft Maxwell software version 5.0 is employed for finite element analysis and verification. Experimental measurements are restricted by available equipment and materials. For instance, the type and precision of measurement apparatuses, the type and dimensions of sensors, and the type and size of multilayer conductor materials all govern the experimental type and measurement accuracy. In contrast, finite element simulation offers the advantage of freely adjusting the sensor size and the thickness and material parameters of multilayer conductors.

Initially, to confirm the efficacy of the proposed analytical model for multilayer conductors composed of diverse materials, the conductivities of the first- and third-layer conductors in the experimental parameters (as shown in Table 1) were modified to σ_6_ = 15 MS/m and σ_8_ = 5 MS/m respectively, while the other parameters remained unaltered. The coil impedance of the T-core coil sensor positioned above three distinct layers of conductors with a hidden hole in the second layer, calculated using Equation (30), is presented in Table 2. Here, the values in the corresponding columns of ε_R_ and ε_X_ represent the relative errors between the analytical calculation outcomes and the finite element simulation results, with the relative errors defined by Equations (48) and (49).

(48) $$\varepsilon_{R}=\left|\frac{R^{analytical}-R^{FEM}}{R^{FEM}}\right|\times 100\%$$

(49) $$\varepsilon_{X}=\left|\frac{X^{analytical}-X^{FEM}}{X^{FEM}}\right|\times 100\%$$

Furthermore, the analytical calculation and finite element analysis of the eddy current density of the conductor were carried out according to the experimental parameters shown in Table 1.

**Figure 7:**
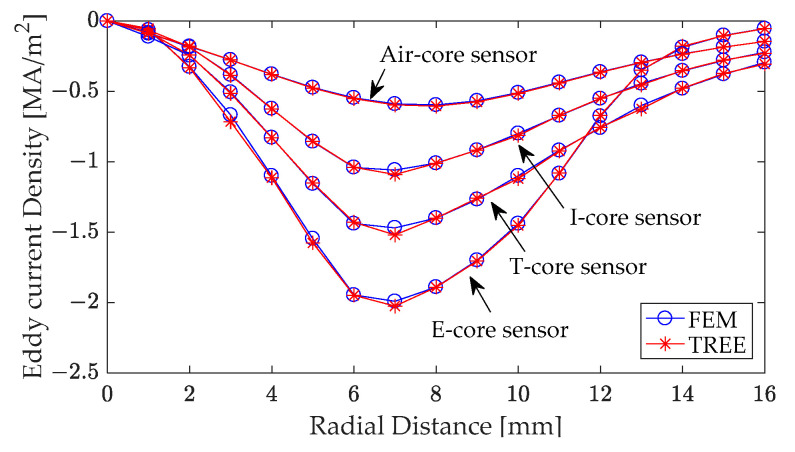
Real component of the eddy current density at a depth of 0.2 mm beneath the surface of the layered conductor, across varying distances from the coil’s central axis.

Figure 7 illustrates the real component of eddy current densities at varying distances from the coil’s central axis. These results were taken at a depth of 0.2 mm below the surface of the layered conductor and are shown for different types of ferrite core sensors. Specifically, the eddy current produced by a T-core sensor within the conductor can be calculated using Equation (29). These computational results demonstrate good agreement with the finite element analysis results.

An analysis of Figure 7 reveals that all core sensor configurations produce the highest eddy current density in the conductor beneath the coil’s inner radius. Furthermore, under identical excitation conditions, the E-core sensor generates the strongest eddy current density within the conductor. The T-core sensor induces a smaller, yet still significant, eddy current density compared to the E-core sensor, but larger than that produced by the I-core sensor. The air-core sensor generates the weakest eddy current density among all sensor configurations.

**Figure 8:**
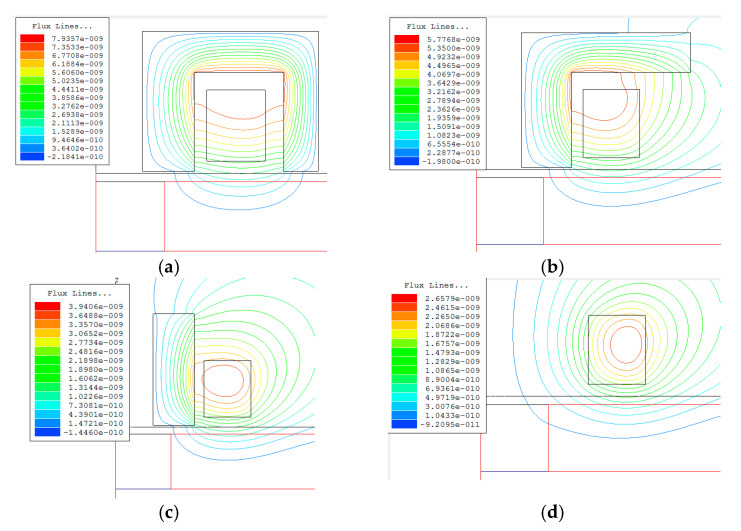
Magnetic flux density distributions generated by sensors of four different core types: (**a**) E-core, (**b**) T-core, (**c**) I-core, and (**d**) air-core. All sensors were positioned above the same layered conductor and subjected to the same excitation.

The magnetic flux distribution generated by each coil type was simulated using Ansoft software version 5.0. Figure 8 depicts the magnetic flux density distributions for the E-core, T-core, I-core, and air-core sensors within the ferrite core, coil, and layered conductor with the embedded hole. The figure highlights a higher concentration of magnetic flux lines within the ferrite core, facilitating the propagation of magnetic flux into the conductor. The E-core configuration exhibits the most efficient magnetic flux transfer path, followed by the T-core and then the I-core. The simulated eddy current density results are consistent with the analytical calculations, providing a rationale for the varying eddy current densities observed in Figure 7 across the different core sensor types.

## 6. Results

**Figure 9:**
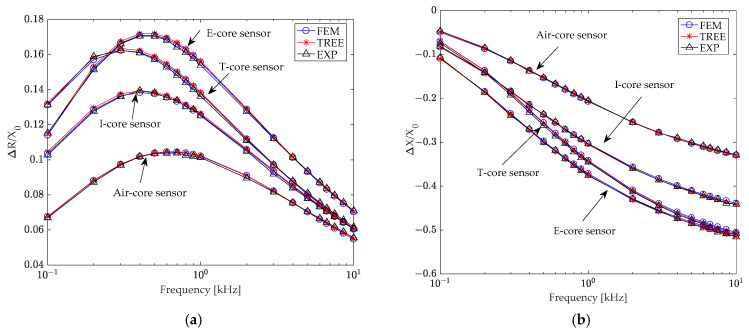
Normalized changes in (**a**) resistance and (**b**) reactance caused by the layered conductor of E-core, T-core, I-core (with a relative permeability of 2500), and air-core (with a relative permeability of 1) sensors, as a function of frequency.

Initially, the multilayer conductor’s conductivities were set to σ_6_ = σ_7_ = σ_8_ = 36 MS/m, and coil impedances (*Z* = *R* + *jX*) were calculated using derived analytical formulas. Subsequently, coil impedances (*Z*_0_ = *R*_0_ + *jX*_0_) were calculated in the absence of conductive material (σ_6_ = σ_7_ = σ_8_ = 0), along with the coil inductance (*L*_0_) of air-core (2.75 mH), I-core (4.93 mH), T-core (7.633 mH), and E-core (10.02 mH) sensors. The changes in coil resistance (Δ*R* = *R* − *R*_0_) and reactance (Δ*X* = *X* − *X*_0_) due to the multilayer conductor were then determined. Figure 9a,b illustrate the normalized changes in coil resistance and reactance, respectively, for different core sensors caused by the layered conductor. Notably, the relative difference between the TREE method and the finite element method remained below 1% in all cases.

Following the calculation of eigenvalues for *m_i_*, *p_i_*, *u_i_*, etc., the impedance of a T-core coil above a multilayer conductor with a hidden defect was determined using a Matlab program based on Equation (30). This computation, performed on a desktop computer (AMD Ryzen 5 5600 G 3.9 GHz CPU (Santa Clara, CA, USA), 24 GB RAM, Windows 10), required approximately 3.869 s under single-frequency excitation. Calculating the impedance for a defect-free multilayer conductor under the same conditions took about 2.535 s. The numerical results achieved a precision of 15 decimal places.

As a verification method, this problem can also be solved using Ansys Ansoft 2D finite element software version 5.0. This involves modeling the multilayer conductor with the defect and the T-core coil sensor, defining excitation and material parameters, and meshing the solution area with 41,714 triangles. The impedance calculation using this method took approximately 15 s. Simulating the defect-free scenario with the finite element method under the same conditions required about 10 s. However, increasing the mesh density or employing a 3D solver would significantly increase the computation time.

When a large number of frequency points need to be calculated, the analytical model offers a substantial advantage over the FEM in terms of computational efficiency. This advantage hinges on the prior calculation of the eigenvalues required for the analytical solution.

Figure 9 reveals that the normalized changes in coil impedance due to the layered conductor vary significantly across different core sensors. This variation primarily depends on the presence and shape of the core. Under identical coil and excitation conditions, the E-core sensor exhibited the highest sensitivity, followed by the T-core sensor, and then the I-core sensor. The air-core sensor demonstrated the lowest sensitivity.

**Figure 10:**
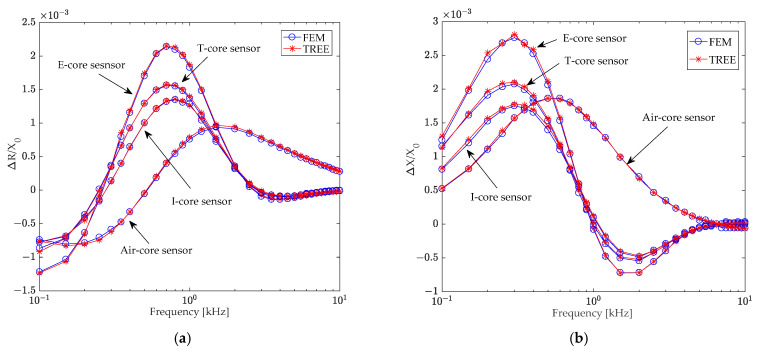
Normalized changes in (**a**) resistance and (**b**) reactance caused by an air hole in the second-layer conductor of E-core, T-core, I-core (with a relative permeability of 2500), and air-core (with a relative permeability of 1) sensors, as a function of frequency.

The impact of an air hole in the second layer conductor on the normalized change in coil impedance was further investigated across varying excitation frequencies, with the results presented in Figure 10.

Figure 10 shows that, due to the small size and location of the air hole within the second conductor layer, the normalized impedance changes for coils with different cores were relatively small and exhibited minimal differences. However, within the excitation frequency range below 1 kHz, the normalized impedance changes decreased in the following order: E-core sensor, T-core sensor, I-core sensor, and air-core sensor.

## 7. The Influence of T-Core Parameters on Coil Impedance Changes

To optimize the design of the T-core sensor for detecting hidden defects in a multi-layer conductor, it is crucial to understand how key T-core parameters, including the air gap radius, upper circular plate radius, column height, and core permeability affect coil impedance. This study employed both an analytical model and finite element simulation to investigate the impact of these parameters on coil impedance. The analysis yielded optimal values for the key T-core parameters, paving the way for the design of a highly sensitive T-core sensor.

### 7.1. Air Gap Radius of T-Core

**Figure 11:**
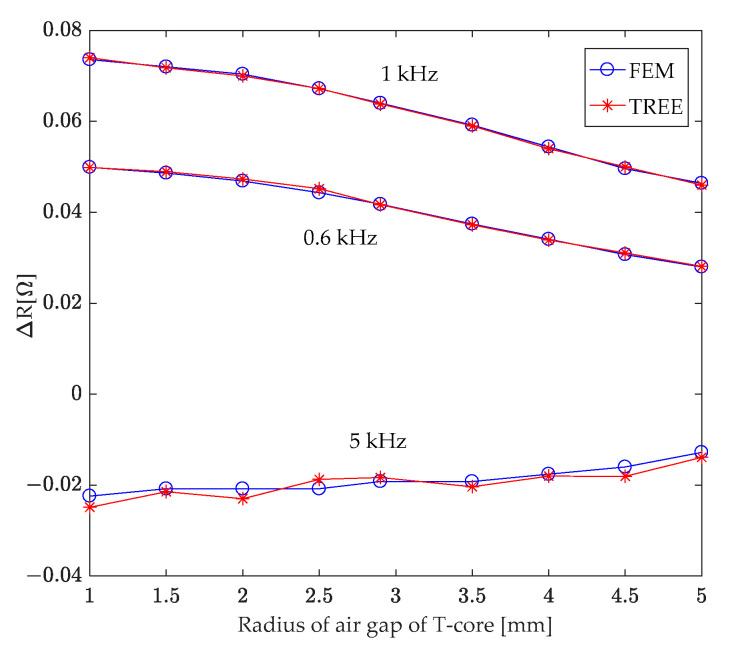
Investigating the correlation between variations in T-core coil resistance caused by a hole in the second-layer conductor and the radius of the T-core air gap.

A T-core sensor with excitation frequencies of 0.6 kHz, 1 kHz, and 5 kHz and an outer column radius (a_1_) of 5.7 mm was investigated. The study focused on the impact of varying the T-core’s air gap radius (a_0_) from 0.1 mm to 5 mm on coil impedance, specifically considering the presence of a second-layer conductor hole. Results indicate that while the air gap radius minimally affects coil reactance, it significantly influences the coil resistance. Figure 11 illustrates this relationship, showcasing the change in coil resistance due to the second-layer conductor hole as a function of the T-core’s air gap radius. Figure 11 reveals that, with a constant outer column radius (a_1_), a smaller air gap radius leads to a greater absolute value of the change in coil resistance caused by the presence of the hole in the second-layer conductor. Within a constant air gap, the variation in coil resistance attributed to the concealed air hole exhibits a dependence on the excitation frequency. The resistance change is most pronounced at 1 kHz, followed by 0.6 kHz, while the least change occurs at 5 kHz.

### 7.2. The Radius of Upper Circular Plate of T-Core

**Figure 12:**
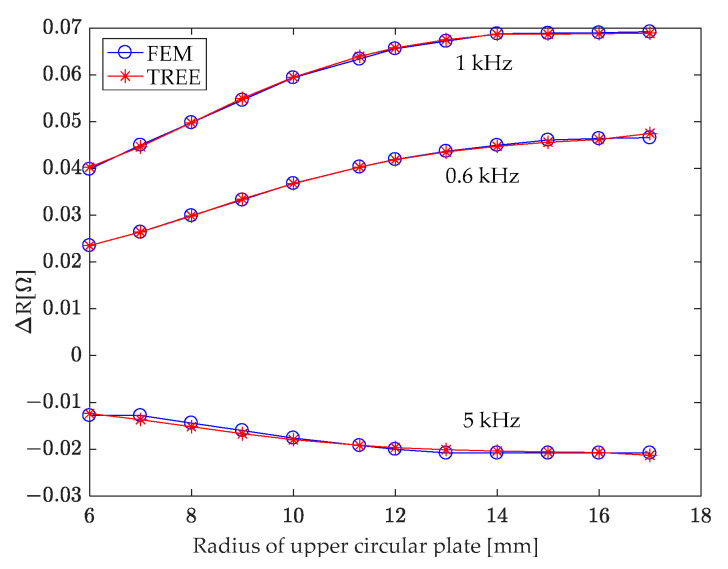
Investigation of the correlation between the variation in resistance of the T-core coil, caused by the presence of a hole in the second-layer conductor, and the radius of the T-core’s upper circular plate.

A T-core sensor, operating at excitation frequencies of 0.6 kHz, 1 kHz, and 5 kHz, was analyzed using the derived analytical model. The radius of the upper circular plate (a_3_) was incrementally increased from 6 mm to 17 mm, while other T-core sensor parameters remained constant (as detailed in Table 1). The study focused on determining the impact of this radius variation on the coil impedance, specifically in the presence of a hole in the second-layer conductor. Calculations revealed that the coil reactance remained relatively unaffected by changes in the upper circular plate radius. Conversely, coil resistance exhibited a more pronounced response, as depicted in Figure 12. This figure shows that the absolute value of the change in coil resistance due to the hole gradually increases with the radius until it reaches 14 mm, after which it stabilizes.

For a constant upper circular plate radius, the variation in coil resistance induced by the hidden hole exhibits a dependence on the excitation frequency. As illustrated in Figure 12, the absolute value of the coil resistance change, caused by the three frequencies, decreases in the order of 1 kHz, 0.6 kHz, and 5 kHz.

### 7.3. Height of T-Core Column

**Figure 13:**
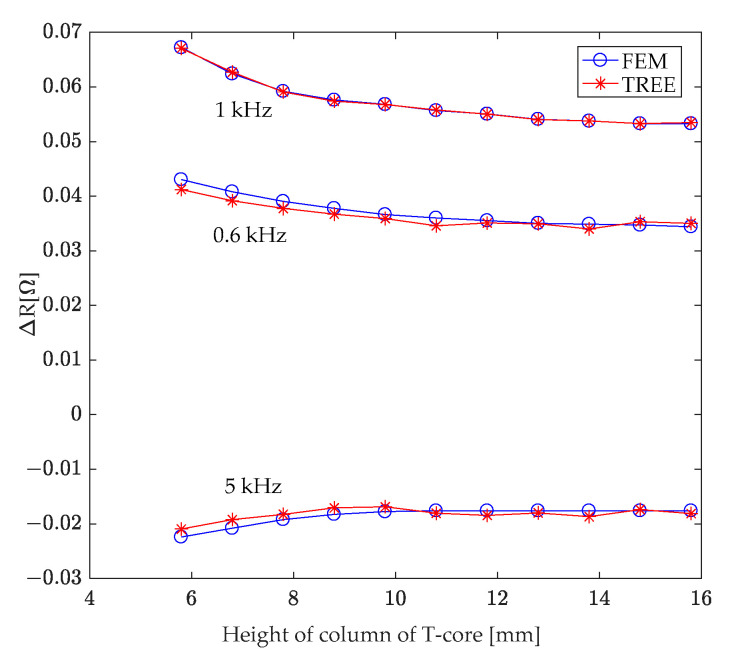
Investigating the correlation between variation in T-core coil resistance caused by a hole in the second-layer conductor and the T-core’s column height.

A T-core sensor was excited at frequencies of 0.6 kHz, 1 kHz, and 5 kHz. The T-core’s parameters remained consistent with Table 1, with the exception of the column height (*h*_1_, as depicted in Figure 2), which was incrementally increased from 5.8 mm to 15.8 mm. Figure 13 illustrates the correlation between the T-core coil’s resistance change and the column height, specifically resulting from a hole in the second-layer conductor. This figure reveals that an increase in the T-core column height leads to a decrease in the absolute value of the coil’s resistance change attributed to the presence of this hole.

While maintaining a constant T-core column height, the variation in coil resistance induced by the hidden hole also exhibits a dependence on the excitation frequency. Notably, the maximum absolute change in coil resistance remains at 1 kHz.

### 7.4. Permeability of T-Core

**Figure 14:**
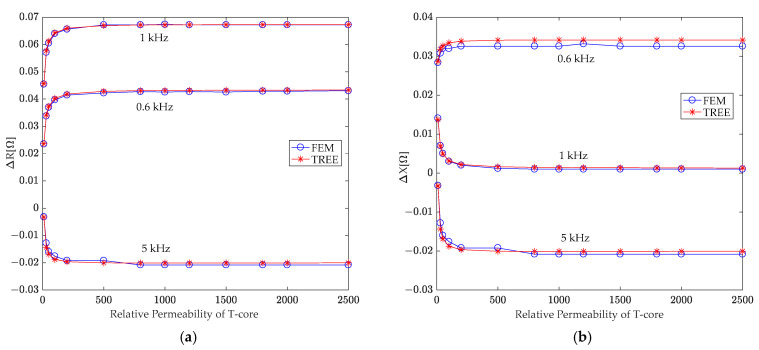
Investigating the correlation between variations in coil (**a**) resistance and (**b**) reactance caused by a hole in the second-layer conductor and the T-core’s relative permeability.

Additionally, the research assessed the effect of the T-core’s relative permeability on the sensor’s sensitivity. The T-core coil sensor was driven by 0.6 kHz, 1 kHz, and 5 kHz sinusoidal currents, respectively. Varying the T-core’s relative permeability from 1 to 2500, the analytical formula (30) was utilized to predict changes in coil resistance and reactance resulting from an air hole in the second-layer conductor. These analytical results were then validated against simulation data obtained using Ansoft Maxwell software, with the findings presented in Figure 14.

Figure 14a demonstrates that as the T-core’s relative permeability increases from 10, the resulting change in the absolute value of coil resistance due to the hole gradually rises, reaching a maximum at a permeability of 500. Further increases in permeability did not lead to a corresponding increase in resistance change. Figure 14b illustrates that increasing the magnetic permeability of the T-core from 10 initially leads to a fluctuating effect on the absolute change in coil reactance due to the hidden hole across various excitation frequencies. Specifically, the absolute change in coil reactance at 0.6 kHz and 5 kHz increases, while it decreases at 1 kHz. Nevertheless, as the permeability rises to 500, these changes in reactance stabilize.

The above study investigated the impact of a hidden hole within a multilayer conductor on coil impedance across three excitation frequencies (0.6 kHz, 1 kHz, and 5 kHz). The analysis explored how variations in key T-core parameters, including the air gap radius, upper plate radius, column height, and relative permeability, influence these impedance changes. Results indicate that, under consistent conditions, the coil resistance change is most pronounced at 1 kHz. Regarding the T-core air gap, minimizing or eliminating the air gap radius is optimal. For the upper circular plate, a radius of 14 mm maximizes the coil impedance change. Regarding the T-core column height, shorter heights are preferable within the constraints of accommodating the coil and ensuring proper contact with the measured conductor, with 5.8 mm identified as optimal in this study. Finally, concerning the T-core material’s relative permeability, a value around 500 was found to be sufficient for achieving the maximum coil impedance change, suggesting that higher permeability does not necessarily translate to better performance.

## 8. T-Core Sensor for Conductor Defect Detection

### 8.1. Crack Detection

**Figure 15:**
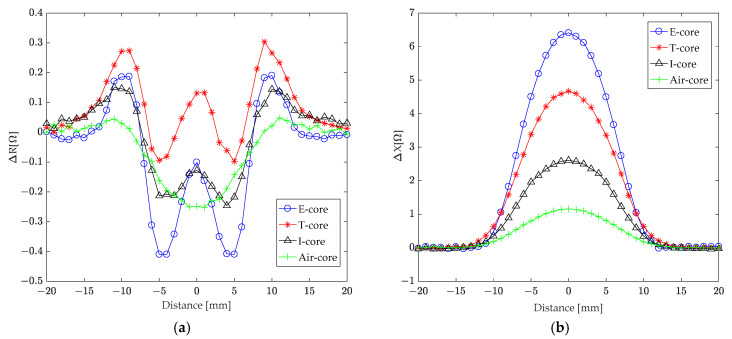
The correlation between variations in coil (**a**) resistance and (**b**) reactance and the distance from the crack’s center line during scanning with various core sensors.

An aluminum plate (30 mm thick) was machined with a 2 mm wide and 10 mm deep rectangular groove to simulate a crack. This simulated crack was then investigated using finite element simulations employing four different ECT core sensors: E-core, T-core, I-core, and air-core. The characteristics of each sensor are detailed in Table 1. The study focused on the changes in coil impedance as the sensors scanned across the crack perpendicular to its direction. Figure 15a,b illustrate the variations in the real (resistance) and imaginary (reactance) components of coil impedance, respectively.

An analysis of the coil resistance changes (Figure 15a) reveals a gradual increase from zero as the sensors approach the crack from 20 mm away from its center line. This increase culminates in peak values at ±10 mm, ±5 mm, and directly above the crack’s center. The resistance changes exhibit symmetry, with values mirroring those observed during the approach when the sensors are moved further away from the crack. Notably, at two of these peak positions (±10 mm), the T-core sensor demonstrated larger resistance changes compared to the E-core, I-core, and air-core coils.

An examination of the coil reactance changes (Figure 15b) shows a minimal initial change as the sensors approach the crack from ±20 mm away from its center line. A significant increase in reactance begins at ±10 mm from the center line, reaching maximum values directly above the crack’s center. Similar to the resistance changes, the reactance variations exhibit symmetry when the sensors are moved away from the crack. While the T-core coil’s reactance changes were smaller than those of the E-core coil, they were larger than those observed for the I-core and air-core coils.

### 8.2. Hole Defect Detection

**Figure 16:**
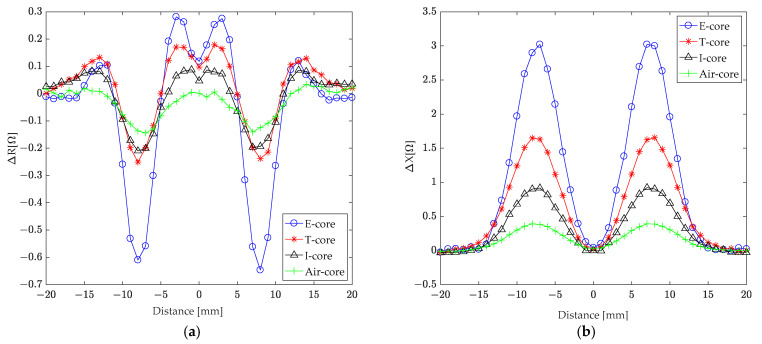
The correlation between variations in coil (**a**) resistance and (**b**) reactance and the distance from the hole’s center during the scanning process of various core sensors through the hole.

An ECT method, simulated using the FEM, was employed to evaluate a hole with a 10 mm depth and 4 mm radius in a 30 mm thick aluminum plate. The simulation investigated the impact of scanning various core sensors (E-core, T-core, I-core, and air-core) through the hole on coil impedance. The core sensor parameters are detailed in Table 1. Figure 16a,b illustrate the changes in coil resistance and reactance, respectively, as the sensors are scanned across different positions relative to the hole center.

An analysis of the coil resistance changes (Figure 16a) reveals that as sensors approach the hole from a distance of ±20 mm, resistance gradually increases from zero, reaching peak values at ±13 mm, ±8 mm, and ±3 mm from the hole center. The resistance changes exhibit symmetry when the sensors move away from the hole. While the T-core coil experiences smaller resistance changes compared to the E-core coil at these peak positions, they are larger than those observed for the I-core and air-core coils.

Figure 16b depicts the changes in coil reactance. As sensors approach the hole from ±20 mm, reactance increases until reaching a maximum at ±7 mm from the hole center, after which it decreases. Directly above the hole, the reactance change is zero. Similar to the resistance changes, reactance changes are symmetrical when the sensors move away from the hole. Though the T-core coil exhibits smaller reactance changes compared to the E-core coil, they remain larger than those of the I-core and air-core coils.

### 8.3. Relationship Between Defect Depth and Frequency Selection

To effectively detect conductor flaws, it is essential to take into account the impact of skin depth on the detection of defects at various depths under different excitation frequencies. A T-core coil sensor was used at various excitation frequencies to examine surface and subsurface cracks (1 mm, 2 mm, 3 mm, and 4 mm below the surface) in a conductor, with the coil axis situated 10 mm away from the crack’s centerline. The finite element method was used to study the changes in resistance and reactance of the T-core coil caused by the cracks at different excitation frequencies.

**Figure 17:**
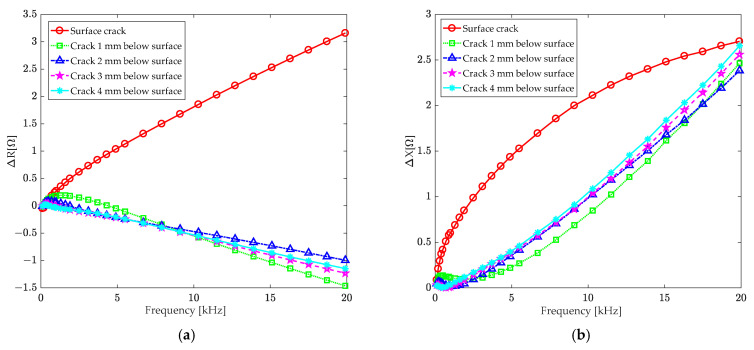
The relationship between the change in (**a**) resistance and (**b**) reactance of the T-core coil and the excitation frequency; the distance between the coil axis and the crack centerline is 10 mm.

**Figure 18:**
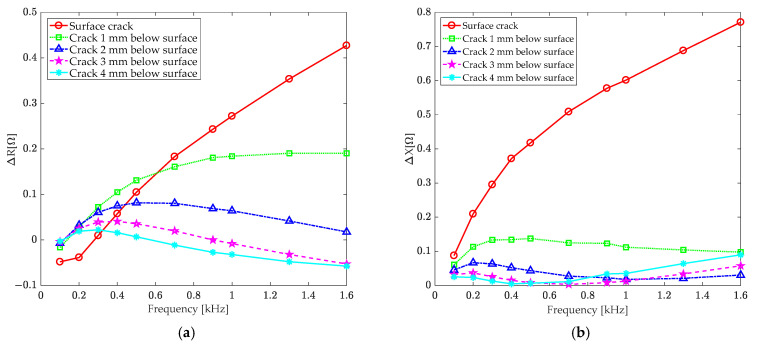
Refinement of the low-frequency area of Figure 17: (**a**) resistance and (**b**) reactance.

Figure 17 displays the results of this study. To better visualize the low-frequency band changes in coil impedance, Figure 18 show a detailed view of the changes in coil resistance and reactance at excitation frequencies below 1.6 kHz.

According to Figure 17, for a surface crack, the changes in coil resistance and reactance increase as the frequency rises. However, for subsurface cracks, the change in coil resistance decreases as the excitation frequency rises. Although the change in coil reactance increases with frequency, there is little difference between cracks of different depths. As illustrated in Figure 18, in the low-frequency excitation range below 1.6 kHz, the coil impedance change decreases as the depth of subsurface cracks rises. However, the distinction between defects of different depths is clearer. Therefore, when detecting subsurface defects, low-frequency excitation should be used to achieve a larger skin depth.

It is worth noting that, as shown in Figure 18, the changes in coil resistance and reactance are more sensitive to deep subsurface cracks at lower excitation frequencies. This suggests that low-frequency excitation is more effective at detecting deep subsurface cracks using the T-core coil sensor.

## 9. Conclusions

This paper presents an analytical model for a novel T-core sensor featuring an air gap positioned above a layered, conductive half-space containing a concealed defect. The TREE method is employed to derive analytical expressions for the eddy current density within the multilayered conductor and the impedance of the T-core coil. These expressions are readily implementable in mathematical software, like Mathematica or Matlab. Validation of the analytical model is achieved through a comparison with FEM simulations and experimental results, demonstrating strong agreement. A comparative analysis reveals that the proposed T-core sensor exhibits a superior flux concentration and shielding compared to an I-core and an air-core sensor, while maintaining a smaller size than an E-core sensor.

The study further explores the relationships between various T-core parameters and the alterations in coil impedance caused by a hidden defect, leading to the identification of optimal values for the primary parameters of the T-core. The advantages of the T-core coil sensor in detecting crack and air hole defects are assessed against other magnetic core and air-core sensors. Additionally, the principles governing frequency selection in the T-core coil sensor and their capability of detecting defects at different depths are discussed.

This analytical model facilitates computer simulations, enables optimized eddy current sensor design, and provides a direct application for conductor defect detection. Future work could extend this solution to analyze more complex conductor geometries and defect shapes.

## Figures and Tables

**Table 1:** The parameters of the coil, T-core, E-core, I-core, and conductor used in experiments, analytical calculation, and FEM.

Core	Air gap radius of core	*a*_0_	2.7 mm
	Inner core radius	*a*_1_	5.7 mm
	Parameter	*a*_2_	10.85 mm
	Outer core radius	*a*_3_	12.85 mm
	Offset	*h*_0_	0.1 mm
	Inner core height	*h*_1_	5.8 mm
	Outer core height	*h*_2_	8.2 mm
	Core relative permeability	*μ*_f_	2500
Coil	Inner coil radius	*r*_1_	6.4 mm
	Outer coil radius	*r*_2_	9.8 mm
	Parameter	*z*_1_	0.7 mm
	Parameter	*z*_2_	4.8 mm
	Number of turns	*N*	400
Conductor	Liftoff	*d*_1_	0.5 mm
	Parameter	*d*_2_	4.5 mm
	Relative permeability	*μ*_6_, *μ*_7_, *μ*_8_	1
	Conductivity	*σ*_6_, *σ*_7_, *σ*_8_	36 MS/m
	Hole radius	*c*	4 mm
	Radius of the domain	*b*	90 mm

**Table 2:** Analytical results of the resistance and reactance of the T-core coil sensor and the relative error with the FEM ones.

f [kHz]	R [Ω]	ε_R_	X [Ω]	ε_X_
0.2	1.57413	0.64%	8.08502	0.024%
1	6.29085	0.94%	31.2587	0.028%
5	19.48457	0.84%	131.7659	−0.12%
10	31.33254	0.82%	249.4438	−0.17%

## Data Availability

Data is contained within the article.

## Appendix A

The matrices T, U, F, G, H, D, G*, H*, V, N, and E were defined in the following form:

(A1) $$\begin{array}{ll} T=[t_{ij}] & =\int_{0}^{a_{0}}rJ_{0}(qr)J_{0}(m^{T}r)dr+\int_{a_{0}}^{a_{3}}rJ_{0}(qr)R_{0}(m^{T}r)dr\\ & +\int_{a_{3}}^{b}rJ_{0}(qr)R_{0}^{\prime }(m^{T}r)dr \end{array}$$

(A2) $$\begin{array}{ll} U=[u_{ij}] & =\int_{0}^{a_{0}}rJ_{1}(qr)J_{1}(m^{T}r)dr+\frac{1}{\mu_{f}}\int_{a_{0}}^{a_{3}}rJ_{1}(qr)R_{1}(m^{T}r)dr\\ & +\int_{a_{3}}^{b}rJ_{1}(qr)R_{1}(m^{T}r)dr \end{array}$$

(A3) $$\begin{array}{ll} F=[f_{ij}] & =\int_{0}^{a_{0}}rJ_{0}(mr)J_{0}(m^{T}r)+\frac{1}{\mu_{f}}\int_{a_{0}}^{a_{3}}rR_{0}(mr)R_{0}(m^{T}r)dr)\\ & +\int_{a_{3}}^{b}rR_{0}^{\prime }(mr)R_{0}^{\prime }(m^{T}r)dr \end{array}$$

(A4) $$\begin{array}{ll} G=[g_{ij}] & =\int_{0}^{a_{0}}rJ_{0}(mr)J_{0}(p^{T}r)dr+\frac{1}{\mu_{f}}\int_{a_{0}}^{a_{1}}rR_{0}(mr)L_{0}(p^{T}r)dr\\ & +\frac{1}{\mu_{f}}\int_{a_{1}}^{a_{3}}rR_{0}(mr)L_{0}^{\prime }(p^{T}r)dr+\int_{a_{3}}^{b}rR_{0}^{\prime }(mr)L_{0}^{\prime }(p^{T}r)dr \end{array}$$

(A5) $$\begin{array}{ll} H=[h_{ij}] & =\int_{0}^{a_{0}}rJ_{1}(mr)J_{1}(p^{T}r)dr+\frac{1}{\mu_{f}}\int_{a_{0}}^{a_{1}}rR_{1}(mr)L_{1}(p^{T}r)dr\\ & +\int_{a_{1}}^{a_{3}}rR_{1}(mr)L_{1}^{\prime }(p^{T}r)dr+\int_{a_{3}}^{b}rR_{1}^{\prime }(mr)L_{1}^{\prime }(p^{T}r)dr \end{array}$$

(A6) $$\begin{array}{ll} D=[d_{ij}] & =\int_{0}^{a_{0}}rJ_{1}(pr)J_{1}(p^{T}r)dr+\frac{1}{\mu_{f}}\int_{a_{0}}^{a_{1}}rL_{1}(pr)L_{1}(p^{T}r)\\ & +\int_{a_{1}}^{b}rL_{1}^{\prime }(pr)L_{1}^{\prime }(p^{T}r)dr \end{array}$$

(A7) $$\begin{array}{ll} G*=[{g*}_{ij}] & =\int_{0}^{a_{0}}rJ_{0}(pr)J_{0}(q^{T}r)dr+\int_{a_{0}}^{a_{1}}rL_{0}(pr)J_{0}(q^{T}r)dr\\ & +\int_{a_{1}}^{b}rL_{0}^{\prime }(pr)J_{0}(q^{T}r)dr \end{array}$$

(A8) $$\begin{array}{ll} H*=[{h*}_{ij}] & =\int_{0}^{a_{0}}rJ_{1}(pr)J_{1}(q^{T}r)dr+\int_{a_{0}}^{a_{1}}rL_{1}(pr)J_{1}(q^{T}r)dr\\ & +\int_{a_{1}}^{b}rL_{1}^{\prime }(pr)J_{1}(q^{T}r)dr \end{array}$$

(A9) $$\begin{array}{ll} V=[v_{ij}] & =F_{1}(vc)\int_{0}^{c}J_{0}(ur)J_{0}(q^{T}r)dr\\ & +J_{1}(uc)vu^{-1}\int_{c}^{b}rF_{0}(vr)J_{0}(q^{T}r)dr \end{array}$$

(A10) $$\begin{array}{ll} N=[n_{ij}] & =F_{1}(vc)\int_{0}^{c}rJ_{1}(ur)J_{1}(q^{T}r)dr\\ & +\mu_{7}^{-1}J_{1}(uc)\int_{c}^{b}rF_{1}(vr)J_{1}(q^{T}r)dr \end{array}$$

(A11) $$E=[e_{ij}]=\int_{0}^{b}rJ_{0}(qr)J_{0}(q^{T}r)dr=\int_{0}^{b}rJ_{1}(qr)J_{1}(q^{T}r)dr$$
